# Entomological surveys and insecticide susceptibility profile of *Aedes aegypti* during the dengue outbreak in Sao Tome and Principe in 2022

**DOI:** 10.1371/journal.pntd.0011903

**Published:** 2024-06-03

**Authors:** Basile Kamgang, João Acântara, Armel Tedjou, Christophe Keumeni, Aurelie Yougang, Anne Ancia, Françoise Bigirimana, Sian E. Clarke, Vilfrido Santana Gil, Charles Wondji

**Affiliations:** 1 Centre for Research in Infectious Diseases, Department of Medical Entomology, Yaoundé, Cameroon; 2 Centro Nacional de Endemias, Ministério da Saúde de São Tomé e Príncipe, Agua Grande, São Tomé e Príncipe; 3 Department of Animal Biology and Physiology, Faculty of Sciences, University of Yaoundé I, Yaoundé, Cameroon; 4 World Health Organization, Port Louis, Mauritius; 5 World Health Organization, Agua Grande, Sao Tome and Principe; 6 Disease Control Department, London School of Hygiene and Tropical Medicine, London, United Kingdom; 7 Vector Biology Department, Liverpool School of Tropical Medicine, Liverpool, United Kingdom; CNRS: Centre National de la Recherche Scientifique, FRANCE

## Abstract

**Background:**

The first dengue outbreak in Sao Tome and Principe was reported in 2022. Entomological investigations were undertaken to establish the typology of *Aedes* larval habitats, the distribution of *Ae*. *aegypti* and *Ae*. *albopictus*, the related entomological risk and the susceptibility profile of *Ae*. *aegypti* to insecticides, to provide evidence to inform the outbreak response.

**Methodology/Principal findings:**

Entomological surveys were performed in all seven health districts of Sao Tome and Principe during the dry and rainy seasons in 2022. WHO tube and synergist assays using piperonyl butoxide (PBO) and diethyl maleate (DEM) were carried out, together with genotyping of F1534C/V1016I/V410L mutations in *Ae*. *aegypti*. *Aedes aegypti* and *Ae*. *albopictus* were found in all seven health districts of the country with high abundance of *Ae*. *aegypti* in the most urbanised district, Agua Grande. Both *Aedes* species bred mainly in used tyres, discarded tanks and water storage containers. In both survey periods, the Breteau (BI > 50), house (HI > 35%) and container (CI > 20%) indices were higher than the thresholds established by WHO to indicate high potential risk of dengue transmission. The *Ae*. *aegypti* sampled were susceptible to all insecticides tested except dichlorodiphenyltrichloroethane (DDT) (9.2% mortality, resistant), bendiocarb (61.4% mortality, resistant) and alpha-cypermethrin (97% mortality, probable resistant). A full recovery was observed in *Ae*. *aegypti* resistant to bendiocarb after pre-exposure to synergist PBO. Only one *Ae*. *aegypti* specimen was found carrying F1534C mutation.

**Conclusions/Significance:**

These findings revealed a high potential risk for dengue transmission throughout the year, with the bulk of larval breeding occurring in used tyres, water storage and discarded containers. Most of the insecticides tested remain effective to control *Aedes* vectors in Sao Tome, except DDT and bendiocarb. These data underline the importance of raising community awareness and implementing routine dengue vector control strategies to prevent further outbreaks in Sao Tome and Principe, and elsewhere in the subregion.

## Author summary

During the first dengue outbreak in Sao Tome and Principe reported in 2022, entomological investigations were undertaken to establish the typology of *Aedes* larval habitats, the distribution of *Ae*. *aegypti* and *Ae*. *albopictus*, the related entomological risk and the susceptibility profile of *Ae*. *aegypti* to insecticides to inform the outbreak response. Surveys revealed the presence of *Ae*. *aegypti* and *Ae*. *albopictus* in all seven health districts of the country with high abundance of *Ae*. *aegypti* in the most urbanised district, Agua Grande. Both *Aedes* species bred mainly in used tyres, discarded tanks and water storage containers suggesting good waste management and improved water supply system could help to reduce *Aedes* densities and the risk of dengue transmission. Analyses also revealed that most of the insecticides tested remain effective to control *Aedes* vectors in Sao Tome, except dichlorodiphenyltrichloroethane and bendiocarb. These findings revealed a high potential risk for dengue transmission throughout the year and underline the importance of raising community awareness and implementing routine dengue vector control strategies to prevent further outbreaks in Sao Tome and Principe, and elsewhere in the subregion.

## Background

**Fig 1:**
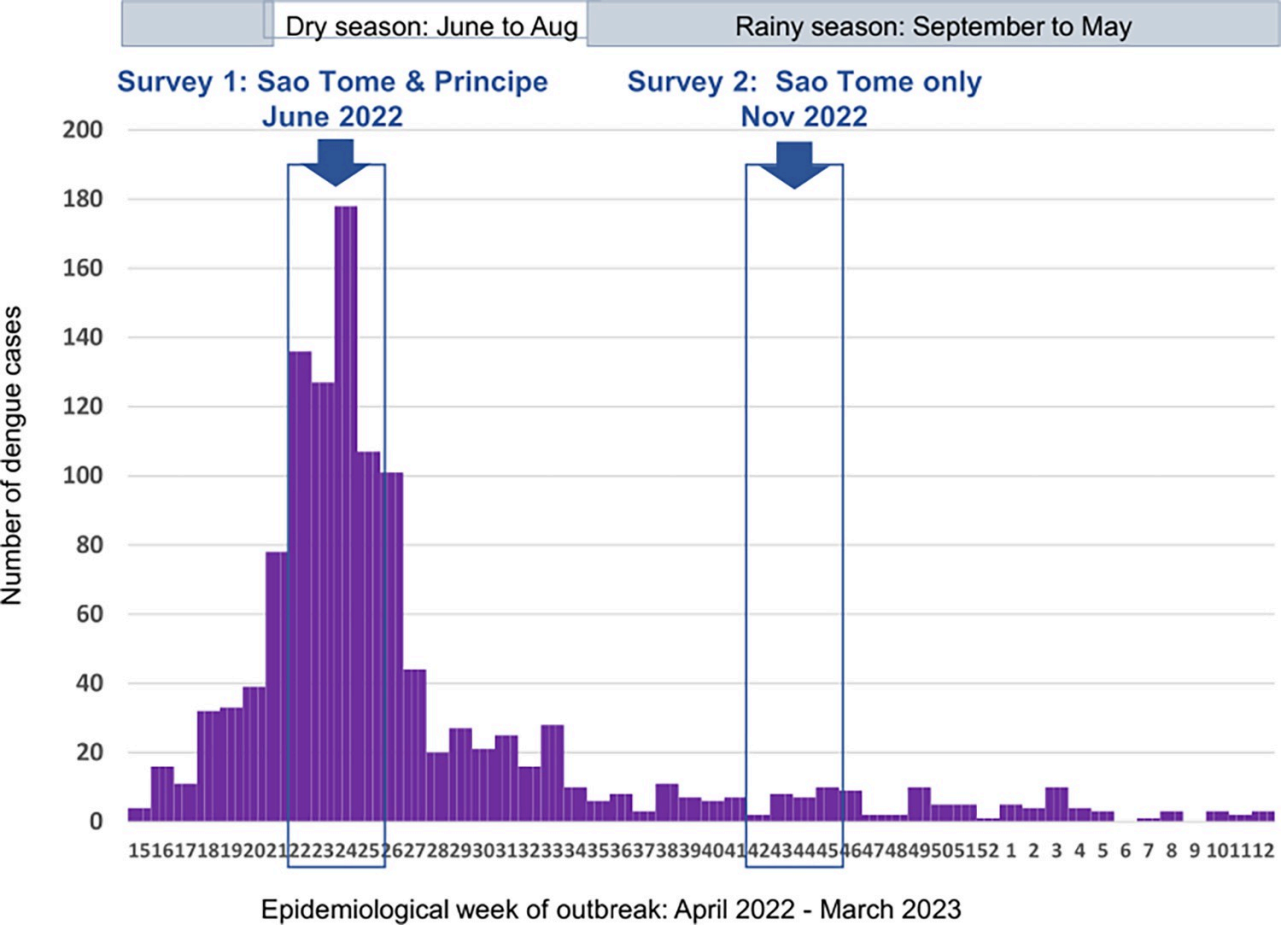
Timing of entomological surveys in relation to cases reported during dengue outbreak in Sao Tome and Principe, 2022–2023.

Dengue is the most important mosquito-borne viral disease in the world in terms of morbidity and mortality. Indeed, one modelling estimate indicates 390 million dengue virus infections per year, of which 96 million (67–136 million) manifest clinically [1]. Dengue is caused by the dengue virus (DENV) belonging to the *Flavivirus* genus and *Flaviviridae* family. This virus is transmitted to vertebrates including humans by the bite of infected female *Aedes* species mosquitoes. Formerly, dengue was considered scarce in Africa probably due to the similarity of symptoms with other infectious diseases like malaria. In countries with endemic malaria, it was therefore often misdiagnosed [2]. However, during the past two decades, increasing numbers of dengue cases have been reported across the continent with major outbreaks in some countries, including Gabon [3], Burkina Faso [4], and Angola [5]. In addition, a study undertaken in Douala Cameroon in 2020 demonstrated that ~13% of acute febrile patients presenting in health facilities in the city are due to dengue [6], suggesting that the dengue prevalence in Africa may be higher than expected. The first ever-recorded case of dengue in Sao Tome and Principe, an island state in Central Africa, was reported in April 2022, reaching a cumulative total of 1,200 cases by March 2023, with the peak of the outbreak in June 2022 (Fig 1). Most cases (68%) were reported in the district of Agua Grande, the most urbanized zone in the island. Since there is no specific treatment nor efficient vaccine against dengue, the control of dengue outbreaks remains reliant primarily on vector control, through the destruction of *Aedes* larval habitats and the application of insecticides to either treat larval habitats or control adults [7]. The implementation of this control strategy requires a good knowledge of the vectors involved in the outbreak.

Both of the vectors most frequently associated with dengue epidemics, *Aedes aegypti* and *Ae*. *albopictus*, are found in Central Africa, but these species have different origins. *Aedes aegypti* originated from African forests [8] whereas *Ae*. *albopictus* originated from South-East Asia forest [9], but has since spread globally and invaded all the continents over the last four decades. This invasive species was first reported in Central Africa in early 2000s in Cameroon [10] and has rapidly spread to almost all central African countries including Sao Tome and Principe where it was first reported in 2016 [11]. Studies performed in Central Africa on larval ecology of both species showed that they colonise the same types of larval habitats composed mainly of used tyres, discarded tanks and water storage containers [12]. However, in the cities where both species are present, *Ae*. *aegypti* has been found to prefer larval habitats located in downtown neighbourhoods with high building density while *Ae*. *albopictus* was mostly found in peri-urban or rural locations surrounded by vegetation [13–15]. The emergence of insecticide resistance in *Aedes* mosquitoes can seriously compromise vector control using insecticides as demonstrated in some countries [16,17]. Data generated in numerous Central African countries reveals significant variation of insecticide resistance according to *Aedes* species tested, the origin of mosquito and the type of insecticide [18–23]. Though to our knowledge, use of insecticide in central Africa is mainly for control of malaria vectors through impregnated mosquito net and indoor spraying, and in agriculture for the protection of crops. Both major mechanisms, metabolic resistance and target site resistance, generally found involved in insecticide resistance in *Aedes* mosquitoes, were suspected as the main causes of resistance in Central Africa. Indeed, knock down resistance (*kdr*) mutations, F1534C, V410L and V1016G were detected in *Ae*. *aegypti* with high frequency for 1534C [20]. Some cytochrome P450 genes were found over expressed in *Ae*. *aegypti* and *Ae*. *albopictus* from Central Africa [20].

In Sao Tome and Principe data on *Aedes* are very scarce. To fill this knowledge gap, this entomological investigation aimed to assess the typology of larval habitats, the entomological risk using Stegomyian indices, the current distribution of *Ae*. *aegypti* and *Ae*. *albopictus* as well as the resistance profile of *Ae*. *aegypti* to insecticides.

## Methods

### Study area

**Fig 2:**
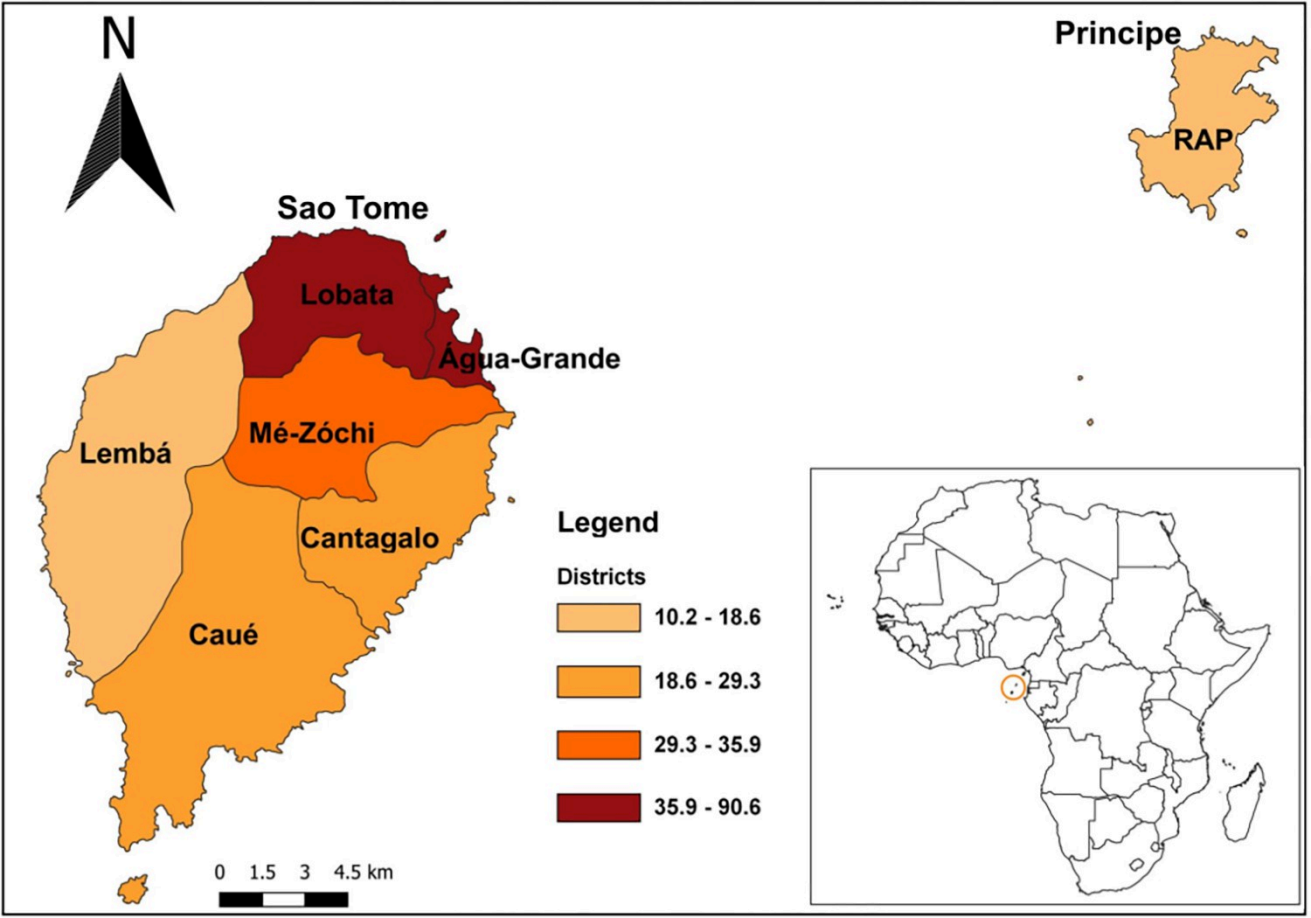
Map of Sao Tome and Principe indicating the seven health districts. Colors indicate population density by health district. The map was created in QGIS version 3.22.12 with the base layer shapefiles downloaded in the publicly available repository GADM version 4.1 (https://gadm.org/download_country.html).

Surveys were carried out in Sao Tome and Principe, an island state of the western equatorial coast of Central Africa. The country consists of two islands, Sao Tome, the bigger island subdivided into six health districts: Agua Grande, Lobata, Me-zochi, Caue, Cantagalo and Lemba, and Principe, the smaller island (RAP: Região Autonóma do Príncipe) comprising one health district (Fig 2). The population of both islands is estimated as 200,000 inhabitants [24]. The climate is humid tropical characterised by two seasons: a long rainy season of nine months duration from September to May and a shorter dry season from June to August; mean annual rainfall is 1,382 mm. Mean temperatures vary a few degrees throughout the year, ranging between 22°C and 26°C. The lowest average temperatures occur during the dry season, while the rainy season experiences higher temperatures(https://climateknowledgeportal.worldbank.org/country/sao-tome-and-principe/climate-data-historical). Agriculture and fishing are the predominant economic activities.

### Sampling and entomological surveys

**Fig 3:**
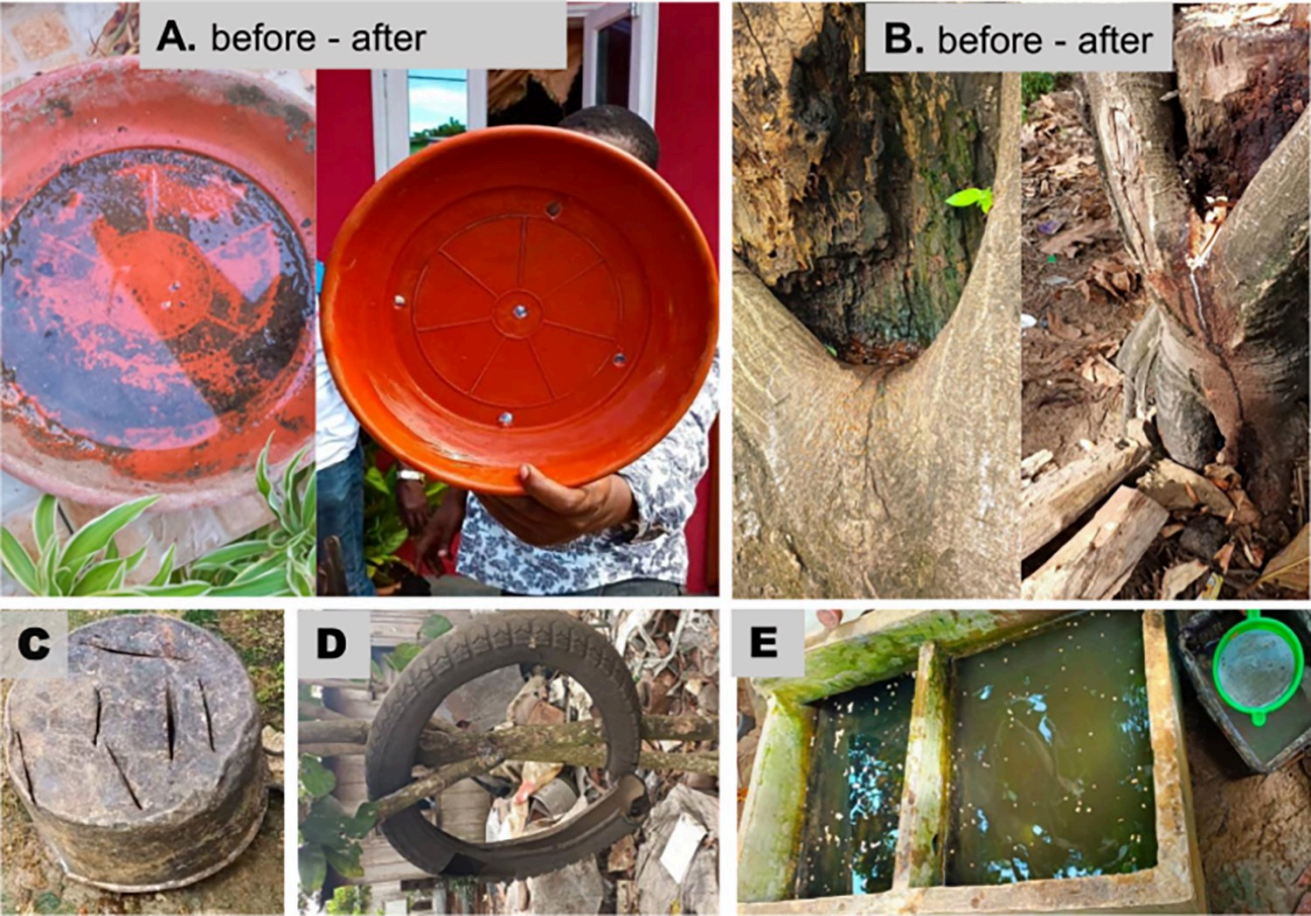
Pictures indicating some examples of action taken on the field during the dengue outbreak to control *Aedes* larvae. A, Destroying plastic saucer of flower pot by perforation with holes; B, Destroying of tree hole (natural larval habitat) by creating drainage channel; C, Destroying of discarded tanks by perforation; D, Destroying used tyres by cutting; E, Water storage container (cement tanks) treated with *Bacillus thuringiensis israelensis* (*Bti*).

Entomological investigations were carried out in June and November 2022, corresponding to the dry and rainy seasons respectively (Fig 1). Nationwide surveys were undertaken in clusters of randomly selected dwellings in each of seven health districts of the country. In each district, a minimum of three clusters were randomly selected; each cluster consisted of 15 dwellings per neighbourhood. During the surveys, each selected dwelling and its surroundings was inspected to record all natural and/or artificial containers with water (potential larval habitats), and number containing at least one larvae or pupae (positive larval habitats). On basis of the nature of the container, source and use of the water, potential larval habitats were classified into three categories: domestic, peri-domestic, and natural. Domestic containers were defined as human-filled receptacles (e.g. storage tanks), while peri-domestic (e.g. discarded tanks, used tyres), and natural receptacles (e.g. rock and tree holes, leaf axils) were those filled by rain [14]. Larvae and pupae found per container were collected and transported to the insectary, isolated from predators such as *Lutzia tigripes*, and reared to adults. Emerged adults were morphologically identified alive using a suitable taxonomic key [25,26]. Adult mosquitoes identified as *Ae*. *aegypti* or *Ae*. *albopictus* from different districts were pooled and reared until obtaining the 2–5 day-old adults of G1 generation used to perform adult bioassays to insecticide. The number of immature stages of each species was estimated from the proportion of emerging adults of each species. During the field investigation all the discarded tanks identified were destroyed and water storage containers were treated with larvicide (*Bacillus thuringiensis israelensis*) (Fig 3). Local entomologists from each health district in Sao Tome and Principe were trained on the identification of *Aedes* larval habitats and their destruction or treatment. Advice was also provided to the population on how to avoid and eliminate *Aedes* larval habitats in their environment.

### Entomological indices

The level of infestation was estimated using traditional Stegomyia indices including Breteau index (BI, the number of positive containers per 100 prospected houses), house index (HI, the percentage of houses infested), and container index (CI, percentage of positive containers). Estimated reference thresholds of HI, BI, and CI established by WHO for dengue and yellow fever transmission risk were applied: whenever HI > 35%, BI > 50, and CI > 20%, the location is considered as high risk of urban transmission of yellow fever virus, whereas HI < 4%, BI < 5 and CI < 3%, is considered to indicate a low risk of disease transmission [27]. Similarly, the categories of low HI < 0.1%, medium HI 0.1%-5% and high HI > 5% were established for classifying risk of dengue transmission [28].

### Insecticide resistance evaluation in *Ae*. *aegypti*

For this study the *Ae*. *aegypti* Benin strain was used as the reference full-susceptible laboratory strain. Bioassays were performed according to WHO protocol using 2–5 days old G1 generation from the field. Four replicates of 20–25 females per tube were exposed to 0.03% deltamethrin, 0.40% permethrin, 0.25% pirimiphos-methyl, 0.05% alphacypermethrin, 0.1% bendiocarb, 1% fenitrothion and 4% dichlorodiphenyltrichloroethane (DDT) for 1 hour. Mortality was recorded 24 hours later and mosquitoes alive or dead after exposure were stored in RNA later or silica gel, respectively. The resistance status was defined as follows: susceptible (mortality rate between 98–100%), probable resistance (mortality rate between 90–97%), and resistant (mortality rate < 90%) [29].

### Adult synergist assay with PBO and DEM

To evaluate the potential role of oxidases and glutathione S-transferases (*GSTs*) in metabolic resistance mechanisms, synergist assays with 4% piperonyl butoxide (PBO) and 8% diethyl maleate (DEM) were performed. Two-to- five-days-old G1 adults from the field were pre-exposed for one hour to PBO or DEM impregnated papers and after that immediately exposed to the selected insecticide. Mortality was scored 24 hrs later and compared to the results obtained with each insecticide without synergist, according to WHO standards [29]. The comparison of mortality rates after pre-exposure of mosquitoes to synergist and without pre-exposure to synergist was performed using Chi-square test.

### Knockdown resistance (*kdr*) genotyping in *Ae*. *aegypti*

Thirty specimens of *Ae*. *aegypti* from G0 selected randomly were genotyped for three different *kdr* mutations: V1016I, V410L and F1534C, chosen because these mutations have been described as involved in pyrethroid resistance of *Ae*. *aegypti* mosquito [30,31]. These mutations have also been previously reported in Central Africa [20]. Based on Moyes et al., review, F1534C and V410L are associated with insecticide resistance and V1016I is associated with insecticide resistance when combined to other *kdr* mutations [31]. Genomic DNA was extracted using the Livak protocol [32], and genotyping of the V1016I, V410L and F1534C mutations was performed by real-time melting curve quantitative PCR [33]. Each PCR reaction was performed in a 21.5 μL volume PCR tube containing 2 μL of DNA sample, 10 μL of SYBR Green (SuperMix), and 1.25 μL of each primer. The amplification conditions were set as follow: 95°C for 3 min, followed by 40 cycles of (95°C for 20 s, 60°C for 1 min and 72°C for 30 s) and then final steps of 72°C for 5 min, 95°C for 1 min, 55°C for 30 s and 95°C for 30 s.

### Data analysis

Categorical variables were summarised by percentages and confidence interval, and numeric variables by means and standard deviations; and compared using the Chi squared and Kruskal Wallis tests, respectively. All statistical analyses were performed using R v4.2.1 and RStudio v2023.03.0 (R Core Team, 2018), and a *p*-value < 0.05 was considered statistically significant.

## Results

### Pre-imaginal infestation

In total we investigated 173 and 241 houses in 22 neighbourhood clusters across six health districts of Sao Tome, during the dry and rainy seasons respectively. In addition, 42 houses in four neighbourhoods were surveyed in Principe (RAP) during the dry season only.

**Fig 4:**
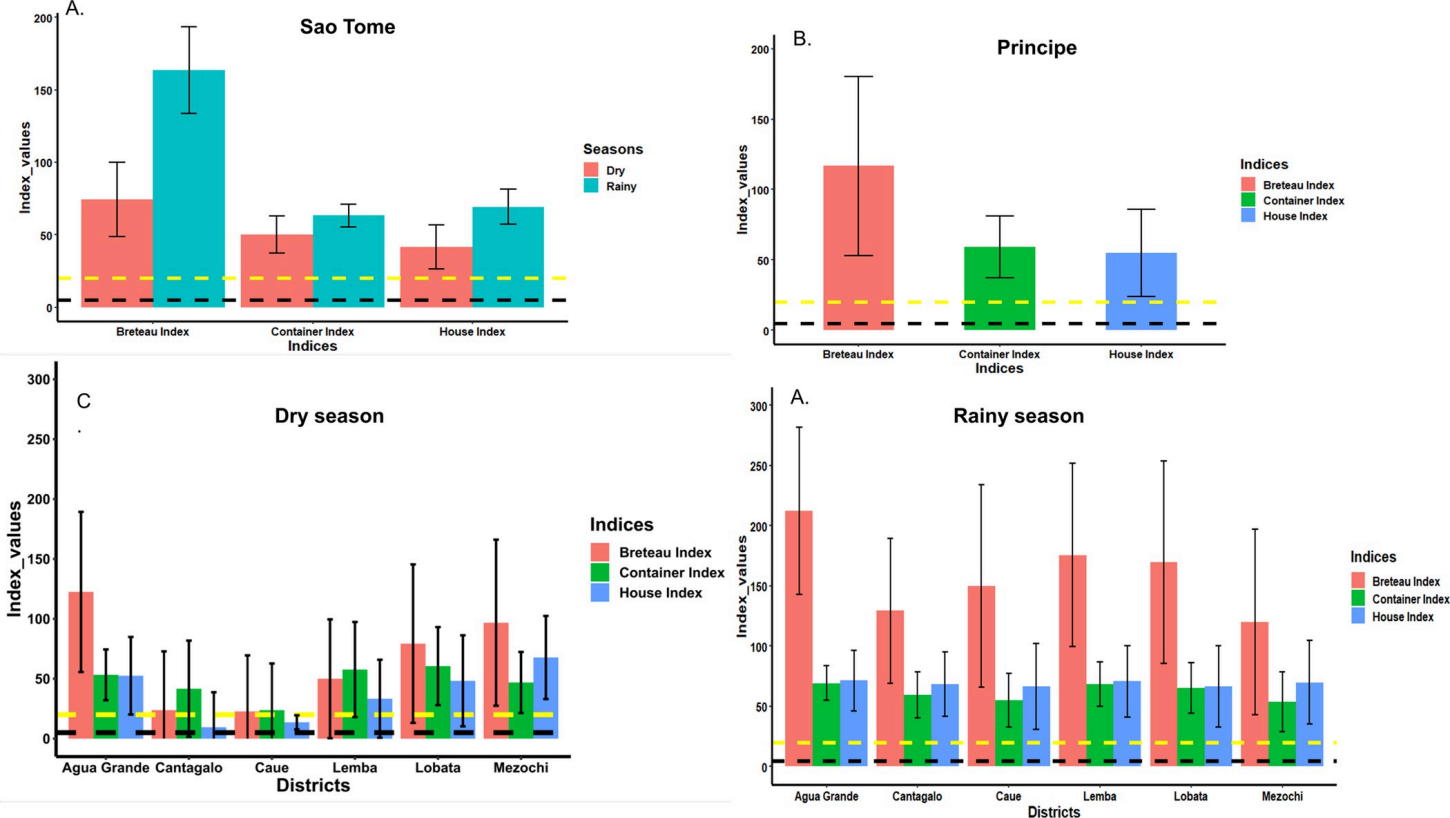
Stegomyia indices calculated in Sao Tome and Principe. A, Indices calculated in Sao Tome according to season. B, Indices calculated in Principe during the dry season. C, Indices calculated during the dry season in Sao Tome per health district; D, Indices calculated during the rainy season in Sao tome per health district.

In Sao Tome, out of 253 potential larval habitats for *Aedes* inspected during the dry season 123 (50.2%) were found positive for *Ae*. *aegypti* and/or *Ae*. *albopictus*, while 385 of 624 potential containers (61.7%) were positive in the rainy season (Table 1). *Aede*s *aegypti* and *Ae*. *albopictus* spp. larvae were sometimes found together with *Eretmapodites chrysogaster*, *Lutzia tigripes* and *Culex* sp. larvae in the same larval habitats. The Stegomyia indices in the dry season were significantly lower than those calculated in the rainy season: house index (HI 41.5 vs 69.3, p<0.0001), Breteau index (BI 74.3 vs 163.5, p<0.0001), and container index (CI 50.2 vs 63.2, p = 0.0005) respectively (Fig 4A and S1 Table). However, in both seasons, all the Stegomyia indices calculated were high and above the thresholds established by WHO to indicate potential high risk for dengue transmission (Fig 4A). When analyses were performed according to location, the house index varied significantly between districts during the dry season (χ^2^ = 28.117, df = 5, *p* < 0.00001) with highest index (HI 67.7) in Mezochi and lowest index (HI 9.5) in Cantagalo (Fig 4C and S2 Table). A similar pattern was observed for the Breteau Index with the highest index (BI 122.5) in Agua Grande and lowest (BI 22.7) in Caue (χ^2^ = 26.064, df = 5, *p* <0.00001). However, no significant difference was found for the container index. When similar analyses were performed for data collected during the rainy season, there was no significant variation in the three indexes between the districts (Fig 4D and S3 Table).

**Table 1:** Container type infested with larvae and or pupae of *Aedes* spp. in rainy and dry seasons in Sao Tome, 2022.

	Dry season (June 2022)				Rainy season (November 2022)			
Container	N inspected	N positive	% positive	Relative contribution: % of positive containers	N inspected	N positive	% positive	Relative contribution: % of positive containers
**Domestic**	**74**	**35**	**47.3**	**27.6**	**164**	**79**	**48.2**	**20.5**
Water storage	48	22	45.8	17.3	130	61	46.9	15.8
Flower pot	13	9	69.2	7.1	23	11	47.8	2.9
Animal drinking bowl	13	4	30.8	3.1	11	7	63.6	1.8
** **								
**Peridomestic**	**175**	**91**	**52.0**	**71.6**	**430**	**283**	**65.8**	**73.5**
Used tyres	67	35	52.2	27.6	163	111	68.1	28.8
Discarded tanks	70	34	48.6	26.8	150	96	64	24.9
Plastic covers	0	0	-	0	29	20	69	5.2
Tin cans	14	5	35.7	3.9	23	12	52.2	3.1
Miscellaneous	24	17	70.8	13.4	65	44	67.7	11.4
** **								
**Natural**	**4**	**1**	**25**	**0.8**	**30**	**23**	**76.7**	**6.0**
Bamboo	0	0	-	0	11	11	100	2.8
Coconut shells	0	0	-	0	7	4	57.1	1.0
Snail shells	0	0	-	0	7	3	42.9	0.9
Tree holes	1	1	100	0.8	2	2	100	0.5
Rock holes	0	0	-	0	3	3	100	0.8
Axil of plant	3	0	0	0	0	0	-	0
**Overall**	**253**	**127**	**50.2**	**100 (n = 127)**	**624**	**385**	**61.7**	**100 (n = 385)**

N inspected, Number of containers with water inspected; N positive, Number of containers found with immature stages of *Aedes* spp.; % positive, Percentage of each type of container found with immature stages of *Aedes* spp.; Relative contribution: Percentage of all positive containers found with immature stages of *Aedes* spp.

In Principe, all three indexes calculated during the dry season (HI = 54.8, BI = 116.7 and CI = 59.0) were high and superior to the threshold established by WHO for potential high transmission risk of dengue (Fig 4B).

### Typology and prevalence of larval habitat

During the entomological investigations in Sao Tome and Principe, all three categories of *Aedes* larval habitats were found: human-filled domestic (water storage container, flower pot and animal drinking bowl, rain-filled peri-domestic (used tyres, tin cans, car wrecks and miscellaneous) and natural (tree holes, rock holes, snail shells, coconut shells, leaf axil of plants). The number of each type of potential container and proportion of containers infested with immature stages of *Aedes* for each island is presented in Table 1 (Sao Tome) and Table 2 (Principe).

**Table 2:** Container type infested with larvae and/or pupae of *Aedes* spp. in dry season (June 2022) in Principe.

Container	N inspected	N positive	% positive	Relative contribution: % of positive containers
**Domestic**	**65**	**37**	**56.9**	**67.3**
Water storage	61	34	55.7	61.8
Flower pot	2	1	50.0	1.8
Animal drinking bowl	2	2	100	3.7
**Peridomestic**	**25**	**18**	**72.0**	**32.7**
Used tyres	12	9	75.0	16.4
Discarded tanks	10	8	80.0	14.5
Miscellaneous	3	1	33.3	1.8
**Natural**	**0**	**-**	**-**	**0.0**
**Overall**	**90**	**55**	**61.1**	**100 (n = 55)**

N inspected, Number of containers with water inspected; N positive, Number of containers found with immature stages of *Aedes* spp.; % positive, Percentage of each type of containers found with immature stages of *Aedes* spp.; Relative contribution: Percentage of all positive containers found with immature stages of *Aedes* spp.

In Sao Tome the most numerous potential larval habitats for *Aedes* spp. inspected were used tyres followed by discarded tanks and water storage containers, independent of the season. These containers were also found to be the most infested with larvae and /or pupae of *Aedes* spp. (Table 1). High infestation rates were observed in all domestic and peridomestic containers inspected on Sao Tome, however the greater number of discarded containers in the peridomestic environment results in these contributing most to *Aedes* vector breeding in the locations surveyed, in both seasons. An increased contribution of natural larval habitats was also noted during the rainy season, with 5.98% of natural containers found to be infested with *Aedes* spp. in the wet season compared to just 0.79% in the dry season. In Principe where inspections were only carried out during the dry season, domestic water storage containers were the most numerous potential larval habitats, and also the main receptacles found infested (61.82%) with immature stages of *Ae*. *aegypti* and/or *Ae*. *albopictus*. This was followed by used tyres and discarded tanks (Table 2).

### Distribution of Aedes aegypti and Ae. albopictus in Sao Tome and Principe

**Fig 5:**
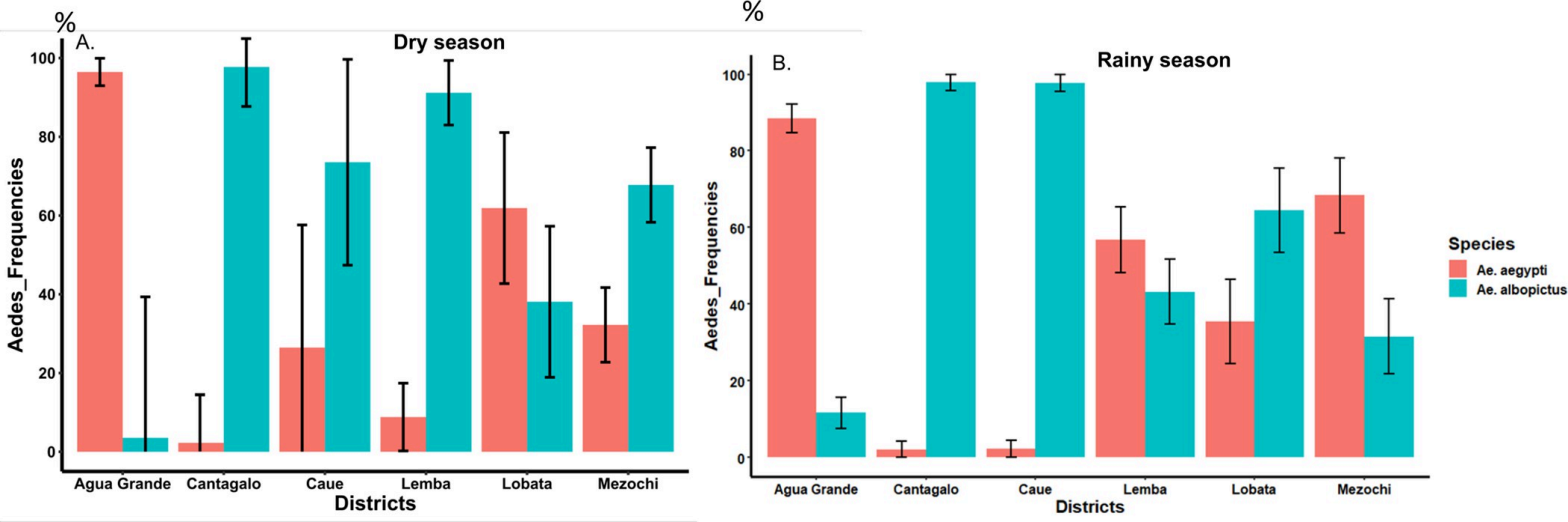
Distribution and prevalence of *Ae*. *aegypti* and *Ae*. *albopictus* per health district. A, dry season; B, rainy season.

In Sao Tome, 1,224 and 2,995 specimens of *Aedes* spp. were morphologically identified during the dry and rainy seasons respectively. These specimens were comprised of 55.47% *Ae*. *aegypti* versus 44.53% *Ae*. *albopictus* (χ^2^ = 2.16, df = 1, p > 0.05) in the dry season, and 53.46% *Ae*. *aegypti* versus 46.54% *Ae*. *albopictus* (χ^2^ = 0.89, df = 1, p > 0.05) in the rainy season. Thus overall, *Ae*. *aegypti* was the most prevalent *Aedes* species in both seasons in Sao Tome. However, when analysed according to the district, *Ae*. *albopictus* was found most prevalent in Caue and Cantagalo irrespective to the season, while *Ae*. *aegypti* was the predominant species in Agua Grande (Fig 5). Nevertheless, in Lemba, Lobata and Mezochi the relative prevalence of the two *Aedes* species did vary according to the season, with a significantly higher prevalence of *Ae*. *albopictus* seen in Lemba and Mezochi during dry season (Fig 5).

In Principe, where investigations were only carried out during the dry season, a total of 126 individuals of *Aedes* spp. were identified comprising 48% *Ae*. *aegypti* and 52% *Ae*. *albopictus*.

### Insecticide resistance profile in *Ae*. *aegypti*

**Fig 6:**
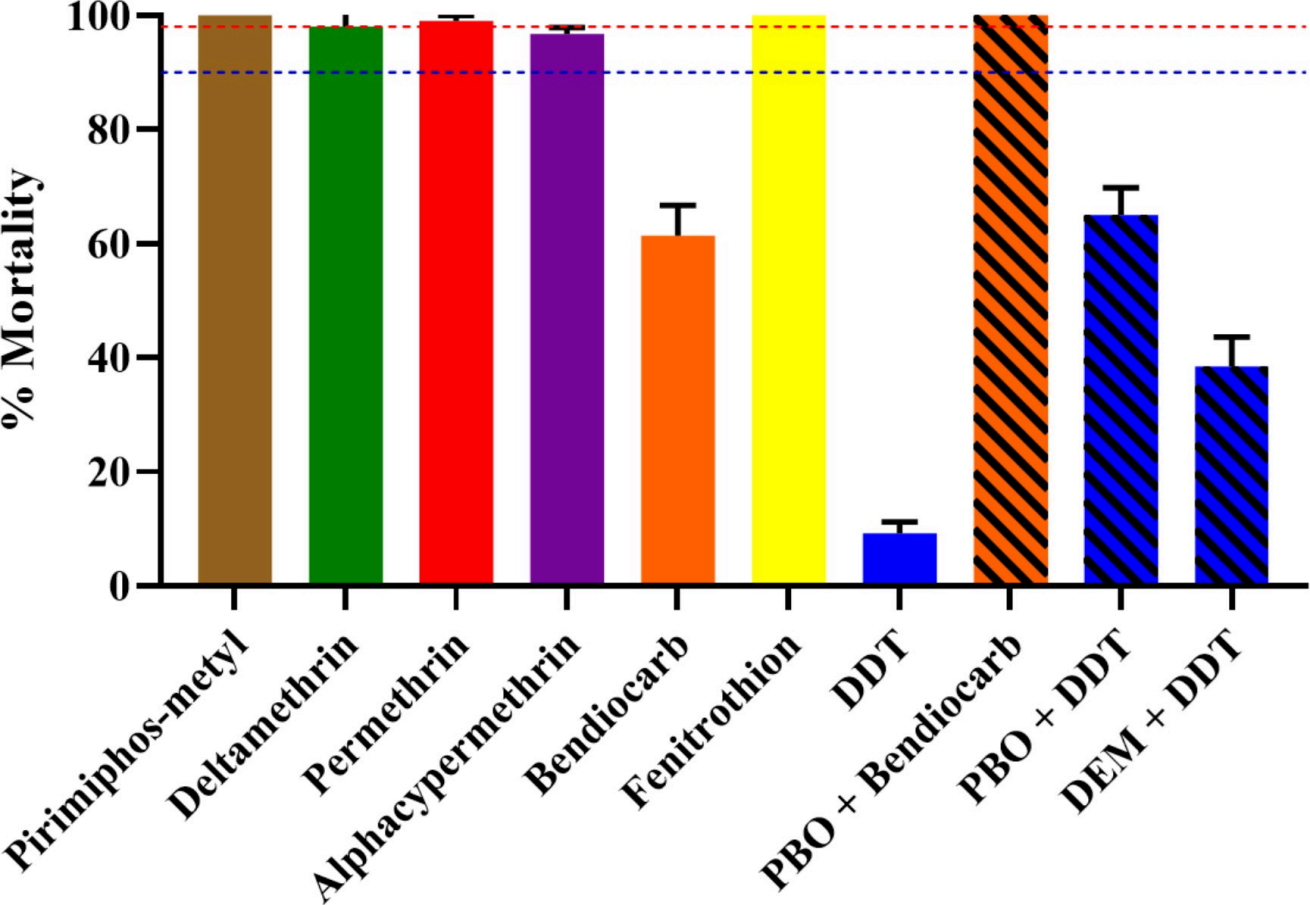
Mortality rates of adult *Aedes aegypti* from Sao Tome when exposed to insecticides alone or with 1 h preexposure to synergist. Error bars represent standard error of the mean. PBO Piperonyl butoxide. DEM, diethyl maleate.

Unfortunately, not enough *Ae*. *albopictus* larvae survived rearing to G1 adults for bioassays. In total 1000 *Ae*. *aegypti* adults from Sao Tome were tested with seven insecticides. The results revealed that this sample was resistant to DDT (9.2% mortality) and bendiocab (61.4% mortality), and probable resistance to 0.005% alphacypermethrin (97.1% mortality), but was susceptible to 0.25% pirimiphos-methyl, 0.03% deltamethrin, 0.40% permethrin, and fenitrothion (with 100%, 98%, 99% and 100% mortality respectively) (Fig 6).

### Synergist assay

Results from synergist assays showed a full recovery of susceptibility to bendiocarb after PBO pre-exposure (61.4±5.3 mortality without PBO pre-exposure vs 100.0 ± 0.0% mortality after PBO pre-exposure, *p*<0.0001). However for DDT, only a strong but not full recovery to DDT susceptibility was observed after pre-exposure to PBO and DEM (9.2 ±2.0 mortality without PBO pre-exposure vs 65.0 ±4.7 mortality after PBO pre-exposure, *p*<0.0001; Fig 4).

### F1534C, V1016I and V410L *kdr* genotyping

Among the 28 specimens of *Ae*. *aegypti* G0 from Sao Tome that were genotyped, no individual was detected with V1016 or V410 resistance mutations, while one heterozygote was found with F1534C resistance mutation.

## Discussion

This was the first study in Sao Tome and Principe to assess the distribution of *Ae*. *aegypti* and *Ae*. *albopictus*, the typology of larval habitats, and the susceptibility profile of *Ae*. *aegypti* to insecticides. The investigations were performed during the first dengue outbreak reported in the country in 2022 [24]. Epidemiological data collected during the outbreak revealed the presence of positive cases of dengue across all the seven health districts of Sao Tome and Principe, however, more than 68% of cases were resident in the health district of Agua Grande [24] which is the most urbanized and most populated area in Sao Tome and Principe.

### Entomological risk and distribution of *Ae*. *aegypti* and *Ae*. *albopictus*

The Stegomyia indices calculated in Sao Tome were considerably higher than the thresholds established by WHO for potential dengue and yellow fever transmission [27], in both seasons. Nonetheless, the values of the Breteau index (BI) obtained during the rainy season were two-times higher compared to the dry season, indicating that the potential risk for dengue transmission is more pronounced during the rainy season. In general BI was highest in Agua Grande compared to other health districts indicative of the high transmission in this location, which has a higher building density. This observation is supported by the epidemiological data across the country [Água Grande (818 cases), Mézôchi (181), Lobata (97), Lemba (20), Caué (23), Cantagalo (47) and Principe (14)] [24]. The entomological data collected during this dengue outbreak show that both *Ae*. *aegypti* and *Ae*. *albopictus* are found throughout the country, though geographical and seasonal differences in their distribution were noted. *Ae*. *albopictus* was found to be the most abundant in four health districts, whereas *Ae*. *aegypti* was the predominant species in Agua Grande. This observation is in accordance with prior data from other locations where both species are sympatric[13–15]. Indeed, studies conducted along an urbanization gradient have demonstrated that *Ae*. *aegypti* prefers urban locations with high building density while *Ae*. *albopictus* is more abundant in rural or peri-urban areas with high vegetation index [34]. This matches with the observations made in different health districts in Sao Tome and Principe. Furthermore, banana trees were found in almost all habitations in the country and could serve as natural larval habitat for *Ae*. *albopictus*. This could explain why, even if *Ae*. *albopictus* was introduced recently as suggested by Reis *et al* [11], this invasive species has become the most predominant species in 4/7 health districts in Sao Tome and Principe. Previous studies in Central Africa have shown that *Ae*. *albopictus* tends to replace resident species *Ae*. *aegypti*, in several locations where both are found sympatric [35–37].

### Typology of larval habitats

The entomological investigation revealed that used tyres, discarded tanks and domestic water storage containers were both the most abundant and the most infested larval habitats for *Aedes* spp. in Sao Tome, irrespective of the season; whereas water storage containers were found to be the principal breeding site in Principe. This difference between the two islands can be explained by deficiencies in the water supply system in Principe, which was more pronounced than in Sao Tome, and led residents to store water in containers for a long period. In fact, some household owners reported they often store water for up to a month in drums, bucket and jerrycans. Socio-anthropological studies could help to better elucidate this phenomenon. The high presence of used tyres and discarded tanks positive for immature stages of *Ae*. *aegypti* and *Ae*. *albopictus* is in accordance with previous data from other Central African cities such as Bangui in the Central African Republic [14], Brazzaville in the Republic of the Congo [38], Yaoundé and Douala in Cameroon [13,15,21,36]. On the other hand, the situation in Principe is closer to what is generally found in Southeast Asia where domestic water storage containers are the most prevalent and productive larval habitats for *Ae*. *aegypti* [39]. In general, these observations suggest that a good waste management system, recycling of used tyres, and communication to support behaviour change could help to reduce breeding sites and the density of *Aedes* in the country. Indeed, during our field surveys advice was given to the population on how to avoid or eliminate *Aedes* larval habitats, all discarded containers that were found were destroyed through perforation, saucers of flower pots were perforated, and large water storage containers (cement tanks) were treated using *Bacillus thuringiensis israelensis* (*Bti*) (Fig 3). This larvicide was chosen because it’s highly specific to Diptera, might be considered a biological control agent and its prior reported effectiveness to control dengue vectors [40–42]. Furthermore, *Aedes* in central Africa have consistently been found susceptible to *Bti [18,22,43]*. *Bti* was also readily available on the islands as it was already being used for *Anopheles* larval control by the malaria elimination programme.

### Insecticide susceptibility profile in *Ae*. *aegypti*

This first study in Sao Tome revealed that *Ae*. *aegypti* is resistant to bendiocab and DDT, but remains susceptible to permethrin, alphacypermethrin, deltamethrin, pirimiphos-methyl and fenitrothion. These findings suggest that all the insecticides tested, except for the two resistant compounds, could be recommended to control *Ae*. *aegypti* in this country. A decreasing susceptibility of *Ae*. *aegypti* population from Central Africa towards DDT, notably in Brazzaville and Yaoundé, was already described in 1970s [44], which may reflect a continuing selection pressure on *Aedes* populations as suggested previously [43]. Indeed, during the last decade DDT resistance has repeatedly been reported in *Ae*. *aegypti* in multiple locations [15,20,21,43,45,46]. A loss of susceptibility was also observed to bendiocarb with moderate level of resistance. Similar results were recently found in Cameroon [23] and Burkina Faso [47,48] in Africa, and several countries outside Africa such as Malaysia [46], Colombia [49], and Mexico [50]. The source of selection driving the observed resistance to DDT and bendiocarb in *Ae*. *aegypti* populations remains unclear, as the programmatic use of insecticides against *Aedes* is limited in the African region [19]. As suggested previously [15,43], domestic use of insecticides through indoor spraying and impregnated bed nets, and agricultural use could be the main sources of resistance selection in *Aedes* vectors in Central Africa, whilst the use of pesticides in agriculture for the protection of crops could promote the emergence of resistance in mosquitoes through contamination of breeding sites and resting places of mosquitoes [15]. During the current outbreak the insecticide used for fumigation was pirimiphos-methyl, an organophosphate, but staff from the National Malaria Elimination Program reported that bendiocarb has been used for indoor residual spraying in Sao Tome in the past. Even though loss of susceptibility in *Aedes* vectors has been reported in other locations in central Africa [19–21], *Ae*. *aegypti* collected from Sao Tome showed a good level of susceptibility to both type I and II pyrethroids tested.

A full recovery of susceptibility to bendiocarb was observed in *Ae*. *aegypti* from Sao Tome after pre-exposure to PBO synergist suggesting that the cytochrome P450 monooxygenases are playing the main role in the observed resistance. On the other hand, only partial recovery of susceptibility to DDT was seen, both after pre-exposure to PBO or DEM synergists. This observation suggests a possible implication of both cytochrome P450 monooxygenases and glutathione S-transferases in DDT-resistant *Ae*. *aegypti* as suspected previously [51]. Among the three *kdr* mutations 1534, 410 and 1016 genotyped only one specimen was found to possesses the 1534C allele, confirming the susceptibility to pyrethroids in *Ae*. *aegypti* in Sao Tome although these results should be treated with caution since the sample size was small and thus sensitivity for detecting resistance mutations is low. These mutations are known to be widely distributed in *Ae*. *aegypti* [31] and have previously been detected in other samples from Central Africa [20,21]. A further limitation of this study was our inability to assess insecticide susceptibility in *Ae*. *albopictus*, due to the low number of specimens successfully reared to G1 adults from larvae collected. Although these data were useful for informing vector control in urban areas, such as Agua Grande where most cases were reported and *Ae*. *aegypti* was the predominant vector, it could be useful to extend this study to obtain a complete picture, since *Ae*. *albopictus* was found to be most prevalent species in four of the other six health districts in Sao Tome and Principe, as well as to continue to monitor insecticide resistance in both species across the country.

## Conclusions

This study has provided for the first time the typology of *Aedes* larval habitats and the distribution of *Ae*. *aegypti* and *Ae*. *albopictus* across Sao Tome and Principe. The results revealed that these species bred mainly in used tyres, discarded tanks and water storage containers. A pattern of susceptibility to insecticides in *Ae*. *aegypti* was established enabling this country to quickly implement insecticide-based control interventions in case of future outbreaks. Indeed, organophosphates notably pirimiphos-methyl was used to control *Aedes* adults in Sao Tome and Principe during this dengue outbreak. Findings generated in this study helped to give advice to the population on practical actions to limit the proliferation of *Aedes* and the Ministry of Health to implement an efficient strategy to control dengue vectors in Sao Tome and Principe. Continued future engagement with political leaders and local communities will also be key to improve systems of water supply and waste management, reducing the number of potential breeding sites in the domestic and peri-domestic environment to reduce the risk of future outbreaks. As there is currently a lack of control programs against arbovirus in many countries in Africa, we recommend using an integrated vector control strategic approach to build upon the successes of well-established vector control program against malaria, to reduce the risk of mosquito-borne disease transmission in general, and to achieve more efficiencies in the fight against mosquito-borne diseases.

## Supporting information

S1 Table Stegomyian indices estimated in Sao Tome per season in 2022. (DOCX)

S2 Table Stegomyian indices estimated in Sao Tome per district during the dry season in 2022. (DOCX)

S3 Table Stegomyian indices estimated in Sao Tome per district during the rainy season in 2022. (DOCX)

S1 Raw Data Entomological data collected in Sao Tome and Principe during dengue outbreak in 2022. (XLSX)
