# Supplementary material for: Entomological surveys and insecticide susceptibility profile of Aedes aegypti during the dengue outbreak in Sao Tome and Principe in 2022
Source: PLoS Negl Trop Dis. 2024 Jun 3;18(6):e0011903. doi: 10.1371/journal.pntd.0011903 (PMC11175431; doi:10.1371/journal.pntd.0011903)
Supplement: S1 Table — (DOCX) [file pntd.0011903.s001.docx]

S1 Table. Stegomyian indices estimated in Sao Tome per season in 2022

| **Index** | **Seasons** | **Value** | **Confidence Interval** | **Test** |
| --- | --- | --- | --- | --- |
| **House Index** | Dry | 41.52047 | 15.25 | **X2=30.499, df=1, p < 0.0001** |
| **House Index** | Rainy | 69.29461 | 12 |  |
| **Breteau Index** | Dry | 74.26901 | 25.48 | **H = 37.959, df = 1, p < 0.0001** |
| **Breteau Index** | Rainy | 163.4855 | 29.94 |  |
| **Container Index** | Dry | 50.19763 | 12.66 | **X2 = 12.16, df = 1, p = 0.00048** |
| **Container Index** | Rainy | 63.24238 | 7.71 |  |

The Kruskal-Wallis test H (non-parametric test for anormal distribution) was used to compare the Breteau index, and the yellow colour indicates a significant variation between indices.
