# Supplementary material for: Entomological surveys and insecticide susceptibility profile of Aedes aegypti during the dengue outbreak in Sao Tome and Principe in 2022
Source: PLoS Negl Trop Dis. 2024 Jun 3;18(6):e0011903. doi: 10.1371/journal.pntd.0011903 (PMC11175431; doi:10.1371/journal.pntd.0011903)
Supplement: S2 Table — (DOCX) [file pntd.0011903.s002.docx]

S2 Table. Stegomyian indices estimated in Sao Tome per district during the dry season in 2022

| **District** | **Season** | **House**  **Index** | **Confidence Interval** | **Container**  **Index** | **Confidence Interval** | **Breteau**  **Index** | **Confidence Interval** |
| --- | --- | --- | --- | --- | --- | --- | --- |
| Agua Grande | Dry | 52.5 | 32.36 | 53.26 | 21.18 | 122.5 | 66.83 |
| Cantagalo | Dry | 9.52 | 29.2 | 41. 67 | 57.17 | 23.81 | 49.03 |
| Caue | Dry | 13.64 | 5.86 | 23.81 | 38.95 | 22.73 | 46.77 |
| Lemba | Dry | 33.33 | 35.53 | 57.69 | 39.73 | 50 | 53.6 |
| Lobata | Dry | 48.28 | 38.02 | 60.53 | 32.58 | 79.31034483 | 66.17 |
| Mezochi | Dry | 67.74 | 34.69 | 46.88 | 25.49 | 96.77419355 | 69.34 |
| **Test** |  | **X2 = 28.117, df = 5, p < 0.00001** |  | **X2 = 9.03, df = 5,**  **p = 0.107** |  | **H = 26.064, df = 5,**  **p = 8.67e-05** |  |
