# Supplementary material for: Entomological surveys and insecticide susceptibility profile of Aedes aegypti during the dengue outbreak in Sao Tome and Principe in 2022
Source: PLoS Negl Trop Dis. 2024 Jun 3;18(6):e0011903. doi: 10.1371/journal.pntd.0011903 (PMC11175431; doi:10.1371/journal.pntd.0011903)
Supplement: S3 Table — (DOCX) [file pntd.0011903.s003.docx]

S3 Table. Stegomyian indices estimated in Sao Tome per district during the rainy season in 2022

| **District** | **Season** | **House**  **Index** | **Confidence Interval** | **Container**  **Index** | **Confidence Interval** | **Breteau**  **Index** | **Confidence Interval** |
| --- | --- | --- | --- | --- | --- | --- | --- |
| Agua Grande | Rainy | 71.43 | 25.0 | 69.19 | 14.29 | 212.5 | 69.41 |
| Cantagalo | Rainy | 68.63 | 26.77 | 59.46 | 18.95 | 129.41 | 60.26 |
| Caue | Rainy | 66.67 | 35.53 | 54.88 | 22.40 | 150.0 | 84.08 |
| Lemba | Rainy | 70.73 | 29.41 | 68.57 | 18.50 | 175.61 | 75.97 |
| Lobata | Rainy | 66.67 | 33.86 | 65.12 | 21.01 | 169.70 | 83.89 |
| Mezochi | Rainy | 70.0 | 34.66 | 53.73 | 24.89 | 120.0 | 76.99 |
| **Test** |  | **X2 = 0.381, df = 5,**  **p = 0.995** |  | **X2 = 9.78, df = 5, p = 0.081** |  | **H = 4.956, df = 5,**  **P = 0.421** |  |
